# The Effects of Substituting Glassware for Plasticware and the Use of an Ethanol Vector on Oocyte Maturation *In Vitro*

**DOI:** 10.1155/2012/914715

**Published:** 2012-03-14

**Authors:** A. D. Macaulay, C. K. Hamilton, P. M. Bartlewski, W. A. King

**Affiliations:** Department of Biomedical Sciences, Ontario Veterinary College, University of Guelph, Guelph, Canada ON N1G 2W1

## Abstract

The intent of this study was to evaluate specific technical aspects of *in vitro* oocyte maturation (IVM), which included container material and solvent delivery vector. Oocytes were matured in oil-free, open-well systems contained in either plastic or glass dishes and compared to control oocytes matured in media droplets on plastic dishes overlaid with mineral oil. Open-well experiments were repeated with ethanol in a quantity sufficient for delivery of nonmiscible compounds. Cleavage rates were significantly decreased in the glassware system when compared to controls. The plasticware open-well system did not differ from either the controls or the glassware groups. Cleavage in glassware with ethanol was significantly lower than controls or plasticware with ethanol. Blastocyst rates were only decreased in the glassware-ethanol treatment when compared to plasticware-ethanol treatment. Cell counts and percentage of TUNEL-positive cells did not differ significantly. Unexpectedly, sex ratio was significantly decreased (34% male) from the expected value of 50% male in the glassware group with added ethanol. The current study demonstrates the sensitivity of IVM to subtle technical changes, resulting in significant developmental consequences.

## 1. Introduction

Appropriate nuclear and cytoplasmic maturation is essential for an oocyte to prepare for fertilization and to develop into an embryo [1]. Embryo production *in vitro* utilizes complex media that coincides to each step in oocyte development: maturation, fertilization, and culture. Common *in vitro* practice exposes cumulus oocyte complexes to a number of substrates including hormones during maturation. Often luteinizing hormone (LH), follicle-stimulating hormone, (FSH), and estradiol are added to IVM media; however, other steroid hormones like androgens [2, 3] or thyroid hormones [4] have been explored because of their classic role in physiology, inducing growth. The contribution of other factors has been explored *in vitro* including biologically derived additives [5, 6], energy sources like pyruvate or glucose [7, 8], and also fully defined media devoid of any unknown biological extracts like serum [6, 9, 10]. Furthermore, the effects of vectors like ethanol (EtOH) [2] and dimethyl sulfoxide (DMSO) [11] which can be used for delivery of poorly water-soluble steroids have been explored. It has been shown that small volumes of EtOH and DMSO (≤1%) do not influence oocyte maturation, but levels of 0.3% or higher can negatively impact blastocyst production [11]. 

Culture media and the components that comprise it play an integral role in appropriate maturation, but the remainder of the system is equally important. Sterile conditions in an incubator with appropriate temperature and gas balance are required for oocyte maturation, fertilization, and embryo culture. Oil overlay of media droplets also contributes to sterility in the media, minimizes evaporation, and limits stress on the oocytes or embryos [12, 13]. Subsequently, the material chosen as the container for the *in vitro* media may have different physical properties that might influence the oocyte maturation. In a similar fashion, additives like estradiol typically need to be delivered in a cytotoxic solvent, and this could also negatively impact oocyte maturation.

Previous reports show that steroid hormones are sequestered by some components that are deemed necessary for oocyte maturation like oil and plastics. Both plastic lab ware [14] and oil overlays [15] have been shown to significantly adsorb steroids from physiological samples or media thus reducing bioavailability. Significant adsorption in other medical related contexts like assay results for glass and plastic for free triiodothyronine, progesterone, prolactin, prostate-specific antigen, and pregnancy-associated plasma protein-A has also been described [16]. For example, 40% of thyroid hormone was found to be adsorbed away from culture media in a previous study [4]. Other reports show that bioactive compounds like biocides from plastic production or an estrogenic xenobiotic, p-Nonyl-phenol, can leach from the container into the media [17, 18]. Some of these concerns have prompted researchers to remove oil from *in vitro* culture systems [19]. A challenge does remain, however, that is, to identify the interactions and effects of the media, oil, solvent, and container.

The purpose of this study was to evaluate subtle changes to embryo maturation conditions by comparing glassware and our lab's standard IVM plasticware as containers for oocyte maturation and also by the addition of a small volume of ethanol to see if there were impacts on embryonic growth and development. It was believed that a change in material as well as the addition of EtOH would negatively impact the ability of the oocytes to mature appropriately, subsequently limiting growth and development.

## 2. Materials and Methods

### 2.1. Experimental Design

Treatments were made at the maturation step only. Our standard maturation protocol (media droplet covered with oil) was carried out for control replicates, which were compared to both open-well plasticware maturation and open-well glassware maturation. The addition of ethanol was explored at IVM only in both the open-well glassware and plasticware systems. EtOH was present at 0.1% in controls and added at 0.2% for EtOH treatments. Eight replicate IVM production groups, completed on different days, contributed to the control group. Each of the treatment groups consisted of seven replicate IVM days. Treatment replicates coincided with control replicates; however, because of limited availability of oocytes all five treatments were not always performed on each day.

### 2.2. Oocyte Collection and *In Vitro* Maturation

Ovaries were collected from a local slaughterhouse (Cargill Meat Solutions, Guelph, Ontario) in a warm (35–37°C) phosphate-buffered saline (PBS) solution (Invitrogen, Burlington, Ontario). Four to five hours after slaughter, oocytes, granulosa cells, cumulus cells, and follicular fluid were collected by vacuum aspiration from follicles between 1 and 8 mm in diameter into Ham's F-10 (Invitrogen, Burlington, Ontario) solution inside 50 mL centrifuge tubes (Fisher Scientific, Ottawa, Ontario) and allowed to settle. The bulk of the fluid was removed, and the remaining sediments mixed with fresh F-10 solution in a petri dish, where good-quality oocytes with homogenous cytoplasm, a complete cumulus cloud with no signs of atresia, and fully grown size greater than 120 *μ*m, were sorted under a stereomicroscope (Nikon, Japan). Oocytes were washed in supplemented TCM-199 (S-IVM) solution twice then washed in filtered (22 *μ*m) S-IVM solution with added hormones. The hormones added to 5 mL of filtered S-IVM are as follows: 10 *μ*L follicle-stimulating hormone (FSH) (Bioniche, Bellville, Ontario) at 82 NIH units/mg diluted 250 ug/mL, 5 *μ*L luteinizing hormone (LH) (Dr. A Parlow, NIH) at 2.3 NIH units/mg diluted to 1 mg/mL, and 5 *μ*L estradiol (E_2_) (Vet. Chiron Co. Guelph, Ontario) at 10 mg/mL dissolved in 5 *μ*L of ethanol. After the hormone wash control-group oocytes were placed 20 per 80 *μ*L droplet overlaid with oil on a 35 mm plastic dish (Nunc, (Fisher) Ottawa). Treatment oocytes were matured in groups of 80 in plastic four-well dishes (Nunc, (Fisher) Ottawa Ontario) (plasticware group) or glass containers (made to specification by Yves Savoret's Glass Shop, University of Guelph) (glassware group) of similar size to the chambers of the four-well plate, in 500 *μ*L S-IVM + hormones. Additionally, ethanol vector doses of 5 *μ*L were added to another set of glassware and plasticware groups. All treatments were incubated for 22 hours at 38.5°C, with a humidified environment of 5% CO_2_.

### 2.3. *In Vitro* Fertilization

After *in vitro* oocyte maturation (IVM) the oocytes were washed twice in sperm-hepes TALP (tyrode's albumin lactate pyruvate) (made in-house with Hepes stock, Invitrogen, Burlington, Ontario) with added bovine serum albumin (BSA) (Cansera, Rexdale, Ontario), and then twice in IVF TALP (made in-house) containing BSA, and heparin (Invitrogen, Burlington, Ontario) for sperm activation. Twenty oocytes were placed in 80 *μ*L drops of IVF TALP under oil in 35 mm petri dishes. Simultaneously, cryopreserved bovine semen (Gencore, Guelph, Ontario) was thawed from liquid nitrogen storage and pipetted into the bottom of a 4 mL tube with 1.25 mL of sperm-hepes TALP. The tubes were incubated at 38.5°C, and the sperms were permitted to “swim up” in the tube allowing the motile sperm to reach the top portion of the fluid, while the immotile and dead sperm remained at the bottom. The sperms were incubated for 60 minutes in the described fashion. After 60 minutes, the top portion of the “swim up” was collected and added to a 15 mL corning tube containing 10 mL of sperm-HEPES TALP (pH 7.3–7.4) and then centrifuged at a low speed (approx 200 ×g) for 8 minutes. The supernatant was removed and 300–400 mL of IVF TALP added to the sperm-containing pellet, agitated and 10 *μ*L of TALP containing approximately 1 × 10^6^ spermatozoa pipetted into each drop. The drops containing both gametes were incubated at 38.5°C in a 5% CO_2_ atmosphere for 18–20 hours.

### 2.4. *In Vitro* Culture

*In vitro* culture was initiated at the completion of fertilization as previously described [3] in synthetic oviductal fluid (SOF)(Chemicon-Millipore, Billerica, Massachusettes) enriched with BSA (1.8%) (Cansera, Rexdale, Ontario), nonessential amino acids (Invitrogen, Burlington, Ontario), essential amino acids (Invitrogen, Burlington, Ontario), sodium pyruvate (Invitrogen, Burlington, Ontario), gentamycin (Invitrogen, Burlington, Ontario), and incubated overnight prior to use. Presumptive zygotes were removed from their fertilization drops and placed into a 15 mL corning tube containing 2 mL of sperm-HEPES TALP. This tube was then vortexed for 90 seconds to strip the cumulus complex from the presumptive zygotes. The denuded presumptive zygotes were then washed twice in sperm-HEPES TALP and twice in SOF media and loaded into SOF drops. 30 presumptive zygotes were placed in a 30 *μ*L drop that was incubated in a tri-gas incubator for 8 days, providing a low-oxygen environment (5% O_2_, 5% C0_2_ 90% N_2_). Embryos were evaluated 48 hours after insemination (hpi) for cleavage rates. Embryo counts were made at day 8 after insemination.

### 2.5. Apoptosis and Cell Counting

**Figure 1:**
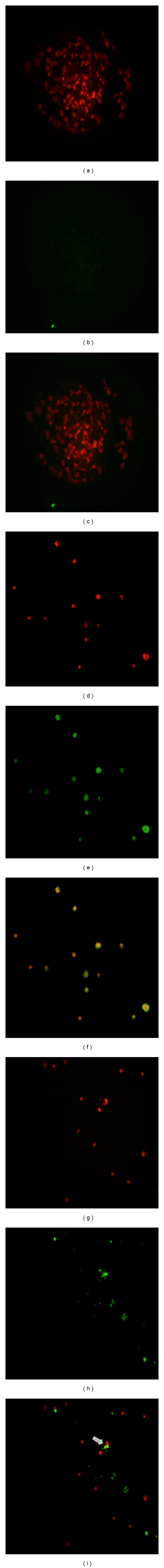
Blastomere nuclear spread (cytoplasm dissolved) stained for cell counting and DNA fragmentation (TUNEL). Apoptosis is detected by green FITC-labeled signals (b, e, h) colocalized with red PI-labeled DNA (a, d, g). Overlay of FITC and PI (c, f, i). Negative control (a, b, c) with strong PI staining for DNA, 100x magnification. Positive control (d, e, f) with strong FITC staining for induced DNA breaks, 200x magnification. Example of treatment staining (g, h, i), 200x magnification. Arrow (i): overlay of red and green signal with fragmentation characteristic of apoptosis.

Collected embryos were immediately mounted on slides and fixed for TUNEL (TUNEL Fluorescein Kit, Roche, Mannheim, Germany) staining and cell counting (Figure 1). Embryos were washed three times in SOF before being exposed for 15 seconds to 0.01 N HCl/0.1% Tween 20 (Cambio, Cambridge, United Kingdom) solution. A few microlitres of HCL/Tween 20 containing embryos were then placed on clean glass Superfrost Plus slides (Fisher, Ottawa, Ontario), subjected to gentle agitation from the tip of an 18-gauge needle, and allowed to dry. The area surrounding the embryo was scored with a diamond pen. Embryos were then fixed with 3 : 1 methanol acetic acid fixative, left to dry for 20 minutes, and then left in fixative overnight. If needed, fixed samples were stored temporarily at −20°C. To begin the TUNEL staining, slides were warmed to room temperature and rinsed in Milli-Q water. Positive controls were treated for 2 minutes in 0.5% pepsin (Fisher, Nepean, Ontario) solution made in 10 mM HCl (Sigma-Aldrich, St. Louis, Missouri) and washed 3 times with PBS. Negative control slides were treated with 50 *μ*L of buffer from the kit. The positive control and treatment slides were treated with a mixture of buffer and enzyme (45 *μ*L and 5 *μ*L, resp., per slide) and covered with parafilm (Pechiny Plastic Packaging, Menasha, Wisconsin). All slides were then incubated for one hour in a humidified chamber at 37°C. After the incubation slides were rinsed twice with Milli-Q water. Propidium iodide (PI) (Sigma-Aldrich, St. Louis, Missouri) was added to the sample, then covered in parafilm, and incubated for 45 minutes at 37°C in a humidified chamber. Slides were rinsed, dried, covered with coverslips, and sealed for viewing under a Leica epifluorescent microscope. PI stained all DNA red, while the nick end labelled DNA was green via fluorescein isothiocyanate (FITC). Overlapping green and red signals produced a yellow signal.

### 2.6. Embryo Sexing by Polymerase Chain Reaction

Embryos used for sexing were first subjected to zona pellucida removal. For this, embryos were removed from culture and washed once in PBS with 0.1% PVA and then transferred into 0.2% pronase solution (Sigma-Aldrich, St. Louis, Missouri). After 2-3 minutes of exposure to pronase, the zonae pellucidae were dissolved, and the embryos were washed three times in SOF. Embryos were vitrified individually, without cryoprotectant, in a 0.2 mL PCR tube that was submersed in liquid nitrogen.

**Figure 2:**
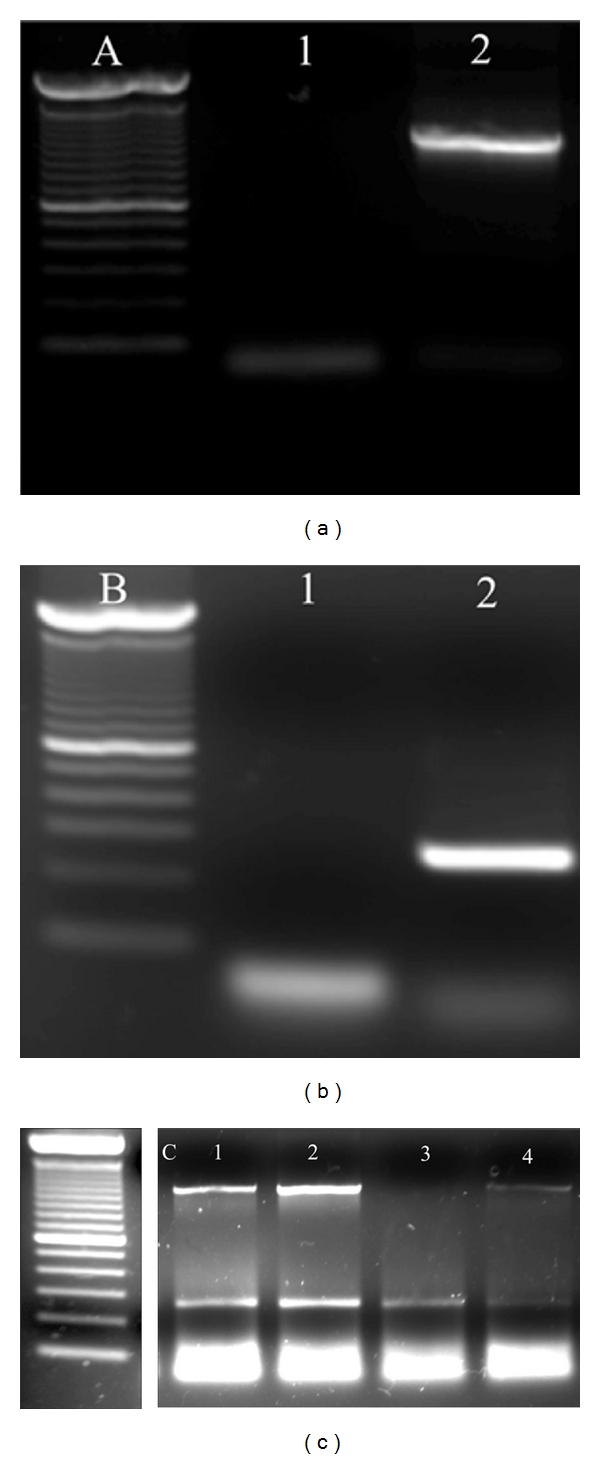
PCR results for embryo sex determination. Example of multiplexed PCR using TSPY and GAPDH. (a) and (b) lane 1: water control with some primer-dimer accumulating below 100 bp. (a) lane 2: TSPY at 1100 bp from a positive control bull testicle sample. (b) lane 2: GAPDH at 260 bp from positive control cattle ovarian tissue. (c) Three males (lanes 1, 2 and 4) and one female (lane 3). All treatment samples are day eight embryos. All ladders are 100 bp ladders.

Zona-free frozen embryos were first lysed by a lysis solution containing 10% protein kinase in a thermocycler for one hour at 37°C, and 15 minutes at 95°C, holding at 4°C at the end. The lysis product was then divided into two halves, one for testis specific protein Y-encoded (TSPY) amplification and the second for glyceraldehyde 3-phosphate dehydrogenase (GAPDH) amplification (Figure 2). The GAPDH amplification was performed using the following program: 94°C, for 10 minutes, and 45 rounds of amplification with denaturation, annealing, and extension for 1 minute each at 94°C, 60°C, and 72°C, respectively. After the cycling, the program ran at 72°C for 10 minutes before holding at 4°C. The primer set for GAPDH (260 base pairs) was: Reverse “5′-ccctgttgctgtagccaaat-3′” and Forward “5′-ctcccaacgtgtctgttgtg-3′”. The TSPY amplification program was as follows: 95°C for 10 minutes and then 40 rounds of amplification, denaturation, and annealing for 45 seconds each at 94°C, 58°C, 72°C, respectively. After completion of these cycles, the program ran at 72°C for 10 minutes before holding at 4°C. The primer set for TSPY (1090 basepairs) was: Reverse “5′-tcttctggtcgctcgtcac-3′” and Forward “5′-ccactgtggttctgggactttg-3′”.

### 2.7. Statistical Analysis

Logit transformation was applied to the data to achieve a normal distribution with equal variance. Analysis was carried out using a mixed linear regression model with SAS 9.2 software (SAS Institute Inc. North Carolina). Values are presented as medians with 95% confidence intervals (C.I.), including the upper limit (UL) and lower limit (LL). The cell count and TUNEL data were analyzed without transformation and were reported as an average with standard error. Confidence intervals (95%) with upper and lower limits are also reported for consistency. Significant differences are reported where *P* < 0.05.

## 3. Results

Cleavage rates (Table 1) for oocytes matured in the glassware group (69.22%) were significantly decreased (*P* = 0.0095) from control oocytes (84.60%). The plasticware group was not significantly different from either control or glassware groups with a cleavage rate of 78.97%. A significant difference in cleavage rate was found between the plasticware + EtOH treatment (81.15%) and the glassware treatments with (69.10%) and without (68.22%) added ethanol (*P* = 0.0151 and *P* = 0.0377, resp.). Blastocyst rates (of oocytes cleaved) were also evaluated (Table 2). No significant differences were found between the controls (34.71%), plasticware (25.77%), and glassware (25.62%) without added ethanol. In plasticware and glassware groups with added ethanol, the glassware group with ethanol (69.10%) was significantly different (*P* = 0.0033) from controls (84.60%) and the plasticware group with ethanol (81.15%) (*P* = 0.0151). Blastocyst rates (percentage of oocytes cleaved) were not significantly different between controls (34.71%) and the plasticware + EtOH (36.23%), but were different between plasticware + EtOH and glassware + EtOH (25.62%) (*P* = 0.0307), differing from the trend of the nonvector groups.

Embryo quality was evaluated at the blastocyst stage by observing sex ratio (Table 3), cell count (Table 4), and percentage of TUNEL-positive cells (Table 5). No significant differences were found between treatments, with controls yielding 48.75% males, plasticware having 55.67% males, glassware having 44.23% males, glassware + EtOH having 34.39%, and plasticware + EtOH having 47.81% males. There was, however a significant shift from the theoretically expected 1 : 1 male : female ratio in the glassware group (95% C.I. = 23.38–47.37) with the additional ethanol (Table 3). As well, cell counts (Table 4) and TUNEL results (Table 5) showed no significant differences between groups. Controls had an average of 99.75 cells of which 3.81% were TUNEL positive, plasticware group blastocysts had 118 cells of which 6.03% were TUNEL positive, and the glassware group had 97.23 cells of which 5.32% were TUNEL positive. In the plasticware + EtOH group cell counts per blastocyst, were 88.33 cells with 4.65% of cells labeled TUNEL positive. Finally, in the glassware group with additional ethanol had cell counts averaging 96.92 cells per blastocyst and 7.30% of cells were TUNEL positive.

## 4. Discussion

The results of this study outline the sensitivity of the *in vitro* developmental environment. A significantly decreased cleavage rate was observed for the glassware group versus the control group, with the plasticware group falling in between. The ethanol treatments resulted in poorer performance for the glassware + EtOH system as cleavage was significantly lower than both controls and plasticware + EtOH. The only significant difference of blastocyst formation was found between the glassware + EtOH and plasticware + EtOH groups. The remaining groups showed no differences in blastocyst rate (percentage of cleaved oocytes). However, where cleavage rates decreased, but where blastocyst rates were unchanged, the percentage of blastocysts of the total collected oocytes placed into the maturation system appears as a decrease in embryo production efficiency. Another finding was that a decrease in sex ratio (i.e., decrease in percentage of males) was observed in the glassware groups with an additional ethanol dose. The developmental rates and indicators of embryo quality for the control group in the present study were consistent with control results previously published by our lab [4]. The control embryo production system did not significantly outperform the open-well plasticware system suggesting that effects of the modified system without the container material change were minimal. This illustrates that simply removing oil from the maturation system is not enough to cause noticeable differences in postfertilization development. However, it appears changing the culture vessel material to glass during oocyte maturation is sufficient to significantly decrease the ability of the oocyte to cleave. There may also be an interaction between the modified glassware system and increased levels of ethanol limiting the proportion of males that develop in the system.

Ethanol is typically limited as a media additive for cytotoxicity reasons [11]; however, if present in low amounts, it may only produce a developmental challenge to the oocyte. Some research suggests a slight challenge to maturation like changes in hydrostatic pressure or oxidative stress which may have positive effects upon the oocytes or embryos [20] and may explain the superior rates of cleavage and blastocyst formation of the plasticware + EtOH group, while the glassware + EtOH group may have simply suffered too much stress that could perhaps have been propagated by the material difference. During maturation, ethanol may limit the ability of the oocyte to mature, fertilize, and develop as an embryo. Depending upon the sex of the embryo, mechanisms of dealing with an ethanol challenge at maturation may be different. Male zygotes tend to develop more quickly in culture and are better responsive to glucose addition to the media [8, 21]. The metabolic differences are believed to be based upon X inactivation [22], as many genes involved in metabolic regulation are found on the X chromosome. A good example of a gene that is X inactivated is glucose 6-phosphate dehydrogenase (G6PDH). Matured COCs do have G6PDH activity [23], and brilliant cresyl blue (BCB) staining [24–26] is used to identify COCs with high metabolic activity. Bovine oocytes that do not metabolize BCB are considered to be of good quality as they are more likely to fertilize and develop to blastocysts. If zygotes are affected by stress at maturation from ethanol (or its immediate derivatives), the activity of G6PDH may aid or limit the potential of the zygote and result in lower blastocyst rates skewed towards one sex. In early female embryos, where X inactivation has not been completed, the two active X chromosomes may provide increased ability to express G6PDH improving embryonic survival, while males with only one X chromosome may have a limited ability to deal with the metabolic stress.

Sex ratio is often studied as an indicator of population health though it is typically reported after birth [27, 28]. Significant shifts in sex ratio have been reported in response to environmental toxicants [28, 29]. A number of studies have reported mammal and nonmammal sex ratio changes, and there has been speculation that this change may be linked to androgens [30–33] as well as estrogens [34]. Several livestock industries are very interested in sex ratio adjustment, particularly dairy because a high proportion of females is desirable for milk production and to replace herd members. One study found that media influenced the sex ratio of developing embryos [35], such that more females were produced. While no mechanism is apparent, a possible stress-related metabolic difference between male and female early embryos could have been responsible for the shift in production [22]. In this study, it is possible that oocytes on the cusp of maturation failed to meet their potential and were eliminated by the added stress of suboptimal culture conditions.

By replacing the material and by removing oil, the properties of the IVM system would have changed. In an open well system there would be concerns for evaporation resulting in the concentration of substrates and metabolites in the media. Evaporation could be influenced by the thermal properties of the different materials affecting heat exchange with the environment. The availability of steroids may have been increased by a material change. No longer would there be adsorption of significant amounts of steroid hormones to plastics or oil in the system that has been previously observed [14–16]. It has been suggested that the mechanisms by which the developing embryo manages stress are acquired later on in development [36]; therefore, the potential stress induced from the altered IVM conditions in this study may be adversely affecting the oocyte/early embryo. Future studies of the impacts of culture materials and vectors and their mechanisms of actions (i.e., steroid assays, evaporation dynamics, transcriptional analyses) would be valuable and could provide further insight into optimal *in vitro *culture conditions.

In the last two decades, scientists involved with *in vitro *embryo production have considered a number of different systems to achieve the best-quality embryos possible such that they would result in healthy offspring. Some labs use one or more of a number of complex systems to sustain growth and development of oocytes and embryos by adding other support cells like fibroblasts, [37] VERO cells (kidney epithelial cells from the African green monkey), [12] and buffalo rat liver (BRL) cells [38] as well as biological extracts like serum, or matrigel, a mixture of basement membrane proteins [39, 40]. Unfortunately, use of these specialized systems confounds our understanding of their function through the release of unknown products from the cocultured cells. There is also concern about disease transmission between these cell types and the parent/offspring involved in the embryo transfer [41]. Subsequently, systems that are fully defined (meaning that all additives and components are known) are being explored and optimized. Most of the focus in this regard is on media components. High purity substrates are used, and biological extracts are eliminated. That being said, in the early 1990s, it was brought to the attention of the scientific community that antioxidants used in the plastic industry were acting as estrogen analogues and leaching from plastics such that cellular proliferation was stimulated, with significant effects on cell culture systems [18] and outlined the need to find ways of managing these factors. While the glassware system may be less optimal now, showing low cleavage rates, and decreased embryo development with added ethanol, it would not be difficult to modify media and additives to suit this material for the efficient production of matured oocytes or embryos. Combined with defined media, less adsorptive glassware would contribute to more controlled media conditions and a better understanding of how the controlled addition of media additives would affect maturing oocyt es.

## 5. Conclusion

Compromised developmental potential was demonstrated through a significant decrease in the cleavage of presumptive zygotes in the glassware treatment groups. The results can be interpreted such that a poor material substitution was made and that glassware was not suitable for effective IVM without further media modifications. The systems, with EtOH vector, showed a significant trend of decreased cleavage in glassware + ETOH compared to plasticware + EtOH and controls, suggesting the main effect on cleavage was a result of using glass as a material. For the most part, normal development of the embryos that cleaved did ensue, the exception being embryos from the glassware + EtOH group. Blastocysts derived from the glassware systems did, however, appear to be of the same quality as those from the other groups based upon TUNEL assay and cell counting. A very interesting shift in sex ratio from the expected 1 : 1 male : female ratio did occur in the glassware group with the EtOH vector as only 34.39% (95% C.I. 23.38–47.37%) of embryos were male. We demonstrated that changing materials could have a negative impact on oocyte maturation and subsequently limit the number of embryos being produced by an *in vitro* system. There may also be a glass-ethanol-linked mechanism limiting male development, a finding that if confirmed and optimized would have a significant impact on industry. We demonstrated the sensitivity of IVM to material and technical effects. The potential still remains for use of glassware as a more inert substance ideal for a defined *in vitro* embryo production system.

## Figures and Tables

**Table 1:** Cleavage rate for control, plasticware, and glassware groups.

	Number of oocytes	Cleavage rate (median)	95% C.I. lower limit	95% C.I. upper limit
Control	1088	84.60^a^	77.47	89.77
Plasticware	863	78.97^ab^	70.16	85.71
Glassware	1113	69.22^bc^	58.48	78.22
Plasticware + EtOH	821	81.15^a^	74.43	86.43
Glassware + EtOH	1570	69.10^bc^	61.33	75.92

Different superscript letters (a, b, c) above values within columns denote significant difference between treatments (*P* < 0.05). Groups with similar letters are not significantly different from each other.

**Table 2:** Blastocyst rate on day eight (% oocytes cleaved).

	Total number of blastocysts	Blastocyst rate (median)	95% C.I. lower limit	95% C.I. upper limit
Control	325	34.71	26.17	44.37
Plasticware	170	25.77	18.80	34.25
Glassware	227	25.62	18.67	34.07
Plasticware + EtOH	255	36.23*	28.81	44.37
Glassware + EtOH	342	25.62*	20.37	31.69

*within columns indicates treatments significantly different from each other (*P* < 0.05).

**Table 3:** Sex ratio determined on day eight embryos.

	Number of sexed embryos	Sex ratio—% male (median)	95% C.I. lower limit	95% C.I. upper limit
Control	249	48.75	27.15	70.82
Plasticware	135	55.67	36.88	72.96
Glassware	80	44.23	23.71	66.93
Plasticware + EtOH	131	47.81	31.09	65.03
Glassware + EtOH	239	34.39*	23.38	47.37

No significant differences between groups. *Denotes significant difference from the expected 50% male : female sex ratio (*P* < 0.05).

**Table 4:** Cell count for day eight blastocysts.

	Number of embryos	Cell count (average)	Standard error	95% C.I. lower limit	95% C.I. upper limit
Control	22	99.75	8.55	81.11	118.39
Plasticware	20	118.39	9.88	96.87	139.91
Glassware	22	97.23	9.88	75.71	118.75
Plasticware + EtOH	21	88.33	9.88	66.81	109.85
Glassware + EtOH	24	96.92	8.55	78.28	115.55

No significant differences between groups.

**Table 5:** TUNEL-positive cells as an indicator of apoptosis.

	Number of embryos	TUNEL-positive rate % (Average)	Standard error	95% C.I. lower limit	95% C.I. upper limit
Control	22	3.81	1.03	1.56	6.05
Plasticware	20	6.03	1.12	3.43	8.62
Glassware	22	5.32	1.12	2.72	7.91
Plasticware + EtOH	21	4.65	1.12	2.06	7.25
Glassware + EtOH	24	7.30	1.03	5.05	9.55

No significant differences between groups.
